# Reproduction Evaluation and Transcription Analysis of *Aphis gossypii* under Various Photoperiods

**DOI:** 10.3390/insects13121105

**Published:** 2022-11-30

**Authors:** Zhe Liu, Shuai Zhang, Ying Zhu, Tianxing Jing, Honghua Su, Jin Hu, Xin Jiang, Yizhong Yang

**Affiliations:** College of Horticulture and Plant Protection, Yangzhou University, Yangzhou 225009, China

**Keywords:** anholocyclic, cotton aphid, photoperiod, survival, reproduction, transcriptome analysis

## Abstract

**Simple Summary:**

*Aphis gossypii* Glover (Hemiptera, Aphidoidae) is a worldwide pest. The life history of *A. gossypii* is complex and there are at least two reproductive strategies in China: holocyclic and anholocyclic types. At present, the strategies of anholocyclic *A. gosspii* for coping with photoperiod and temperature conditions in autumn and winter remain unknown. Therefore, we studied the reproduction pattern of anholocyclic *A. gossypii* under different light conditions at low temperatures and performed a transcriptional analysis of them. In this study, the results showed that changes in photoperiod affect the reproduction of *A. gossypii*. High-throughput sequencing analysis revealed that photoperiodic changes lead to differentially expressed genes (DEGs) in corresponding pathways. Through screening, we found that these DEGs were concentrated in Hsp70 family genes, legumain-like genes, and cathepsin B-like genes. This suggested that these DEGs play an important role in photoperiod changes that regulate the reproduction of *A. gossypii*. Our results will provide insights into control populations of *A. gossypii* through the regulation of reproduction.

**Abstract:**

*Aphis gossypii* Glover (Hemiptera, Aphidoidae) is a polyphagous pest, whose complex phenotypic form, combined with its high fecundity and short reproductive cycle, has caused serious economic losses to agriculture worldwide. Photoperiod plays an important role in the reproduction of aphids. However, the molecular mechanisms underlying its response to seasonal photoperiodic changes are not known. In this study, the effects of different photoperiod treatments (8 L:16 D, 10 L:14 D, 12 L:12 D and 14 L:10 D) on *A. gossypii* reproduction in the first, third, and fifth generations at low temperatures were investigated. Then, transcriptome sequencing analysis was performed after the fifth generation of *A. gossypii*, exposed to different photoperiods (8 L:16 D and 12 L:12 D), using high-throughput sequencing technology. The results showed that (I) the effect of photoperiod on aphids was gradually evident with increasing exposure generations. In general, daylight extension help the *A. gossypii* to reproduce with the optimum photoperiod of L:D 12:12. (II) The transcriptome analysis results showed that 170 differentially expressed genes (DEGs) (123 downregulated and 47 upregulated genes) were identified between aphids under 8 h daylight and 12 h daylight. (III) Gene Ontology (GO) enrichment analysis showed that the DEGs involved in “proteolysis”, “metabolic process”, “peptidase activity” and “structural molecule activity” were significantly enriched; Kyoto Encyclopedia of Genes and Genomes (KEGG) enrichment analysis showed that there were more DEGs in “Longevity regulating pathway-multiple species”, “Lysosome”, “Endocytosis”, “Spliceosome” and “Protein processing in endoplasmic reticulum”. (IV) Ten related genes were chosen for validation of statistical analysis based on RNA-Seq by the reverse transcription quantitative (RT-qPCR). The comparison was consistent with the expression pattern and supported the accuracy and reliability of RNA-Seq. In summary, the genes involved in these pathways play an important role in the reproduction of *A. gossypii* under photoperiodical changes. These will contribute to the sustainable management of cotton aphids through the disruption of their reproduction by the method of RNA interference in the future.

## 1. Introduction

In temperate regions, the length of day and night variations in different seasons are detected by most organisms and are generally used as a signal to calculate environmental changes [1]. Photoperiod is a relatively stable environmental factor that influences insect growth and development, reproduction, physiological metabolism, behavioral rhythms, and population [2,3,4]. Some insects, such as *Riptortus clavatus, Migratory locusts*, etc., receive photoperiodic signals to survive adverse environmental infestations in the future by diapause [5,6,7]. Another group, particularly the aphid species, finds that the shortened photoperiod induces a reproductive shift to survive the cold winter. Currently, the photo periodically induced reproductive transformation of aphids is known for *Acyrthosiphon pisum*, *Aphis gossypii*, *Aphis fabae*, *Myzus persicae,* and *Megoura viciae* [8,9,10,11,12].

*Aphis gossypii* (cotton or melon aphid) (Hemiptera, Aphidoidea), an economically important destructive pest, colonizes more than 900 species of host plants, including cereal crops, *Brassica* crops, Malvaceae plants, Cucurbitaceae plants, and some fruits [13]. In addition, as a sap-sucking pest, cotton aphids can transmit 76 viruses, as well their honeydew can also induce sooty blotches, causing significant economic losses to agricultural and forestry production worldwide [14]. Studies have reported that cotton aphids can have at least two reproductive strategies: holocyclic and anholocyclic types [15,16]. The former produces sexual aphids in late autumn or early winter and overwinters with fertilized eggs, while the latter engages in parthenogenesis throughout the year. In recent years, a large number of studies have focused on the sexual phase of cotton aphids [17,18]. The reproductive strategy of asexual reproduction clones (anholocyclic) under seasonal photoperiodic changes in autumn and winter is relatively lacking. However, these overwintering generations may provide a substantial source of the population for spring crops. This study will complement the pattern of production of the anholocyclic *A. gossypii* under photoperiodic change, help to reveal the reproductive responses of photoperiod to this population, and provide a theoretical basis for sustainable pest control development.

In this study, we determined for the first time the effect of photoperiod on the fecundity of anholocyclic *A. gossypii.* Then, differentially expressed genes (DEGs) between 12 h and 8 h aphids under daylight conditions were identified by an RNA-seq approach, followed by reverse transcription quantitative PCR (RT-qPCR) to validate the transcriptome results. The DEGs and significantly enriched signaling pathways of aphids reared under different photoperiods were then discussed. Our study aimed to (I): examine the effect of photoperiod on the reproduction of *A. gossypii*; and (II): provide a molecular basis for photoperiod adaptation in *A. gossypii*. These results interpreted the changes in aphids under photoperiodical variation from a molecular ecological perspective and provided a theoretical basis for the future reduction of their population base of overwintering aphids by RNA interference.

## 2. Materials and Methods

### 2.1. Aphid Maintenance

Apterous viviparae *A. gossypii* colony was collected originally in cotton plants in Yangzhou, Jiangsu Province, China (32°23′25″ N, 119°25′20″ E), in the spring of 2020. Biotypically identified as cotton-type. Test insects were reared on cotton seedlings at the Yangzhou University under an artificial climate chamber for more than 10 generations prior to the start of the study (26 °C ± 1 °C, 65% relative humidity, 12,000 Lux light intensity, and 14 L:10 D photoperiod). Aphid population densities were monitored frequently to avoid the emergence of winged aphids. The host plants used to maintain aphid populations were SGK321 transgenic cotton provided by the Institute of Plant Protection, Chinese Academy of Agricultural Sciences, and cultured to the 2-leaf stage for the experiment.

### 2.2. Ecological Characteristics of A. gossypii

To examine the effects of photoperiod on the development of *A. gossypii*, three to four adults were allowed to lay their young for 24 h and then removed. About 6–7 offspring were retained on one single plant in mini clip-cages and reared under 18 °C, 65% relative humidity, and 12,000 Lux light intensity with four different light–dark cycles (8:16, 10:14, 12:12, and 14:10), respectively. Adult fecundity of the first, third, and fifth generations under each photoperiod treatment was calculated. There were 4 to 5 replicates for each treatment. To further verify the effect of photoperiod on cotton aphids, we selected the fifth generation of aphids with a photoperiod of 8:16 and divided them into two treatments, under continuous short light (8:16) and shifting to long light (12:12) and assessed the fecundity of these adults after two generations.

### 2.3. RNA Sequencing Sample Collection

Based on the results of the effect of photoperiod on fecundity, we selected the 5th generation of aphids and collected samples under 8 h of short light and 12 h of long light, where the differences were the greatest. To eliminate or reduce the potential effects of embryos in the adult ovaries, 4th instar nymphs were snap-frozen in liquid nitrogen and stored at −80 °C in an ultra-low temperature refrigerator (Thermo Scientific, Waltham, MA, USA). There were three biological replicates per treatment in RNA-seq. Each biological replicate contained 200 4th instar wingless aphids, respectively.

### 2.4. Transcriptome Assembly and Gene Annotation

Total RNA was extracted from the whole bodies of the treated aphid samples using an RNA-easy^TM^ Isolation Reagent (Vazyme, Nanjing, China) according to the manufacturer’s protocol. The QC (Quality Control) approach focused on the precise detection of RNA integrity by the RNA Nano 6000 Assay Kit of the Bioanalyzer 2100 system (Agilent Technologies, Santa Clara, CA, USA). Illumina library generation was completed at Beijing Novegene Technology Co., Ltd. (Beijing, China). Six libraries were built using light lengths of 8 and 12 h. After sequencing to obtain raw reads, adapter sequences and low-quality reads were filtered to obtain clean reads. HISAT2 software [19] was used to compare clean reads with the reference genome (https://bipaa.genouest.org/sp/aphis_gossypii/download/ accessed on 1 March 2021), and to obtain the localization information for the reads. Mapped reads for each sample were assembled by StringTie (v1.3.3b) [20] and Featuv1.5.0-p3 was used to count the number of reads for each gene.

### 2.5. Identification of DEGs

Differential expression analysis was performed using the DESeq2 R package (1.20.0 https://bioconductor.org/ accessed on 1 March 2021) for both conditions. Genes identified by DESeq2 with adjusted *p* values of less than 0.05 (as calculated by the edge R package (3.22.5 https://bioconductor.org/packages/release/bioc/html/edgeR.html accessed on 1 March 2021) were designated as differentially expressed. GO enrichment and significantly enriched KEGG pathways were identified through cluster Profiler R package for the analysis of DEGs. The local version of the GSEA analysis tool (http://www.broadinstitute.org/gsea/index.jsp accessed on 12 March 2021), GO and KEGG datasets were used for GSEA (Gene Set Enrichment Analysis) independently.

### 2.6. Validation of RNA-Seq Data by RT-qPCR

The same RNA samples used in transcriptome sequencing (*A. gossypii* living in an 8:16 and 12:12 photoperiod for 5 generations) were used to assess the reliability of sequencing and analysis via RT-qPCR. Following that, we validated the expression of candidate genes (in the continuous 8:16 light–dark cycles) and (switching from the fifth generation to a 12:12 light–dark cycle) treatment. A total of 1% agarose gel electrophoresis was used to confirm the integrity of the RNA. In addition, the RNA concentration was assessed using a NanoDrop2000 Ultra-micro nucleic acid protein analyzer (Thermo Scientific, Waltham, MA. USA). Ten DEGs screened according to the transcriptome data were selected for RT-qPCR, and *agos*DIMT and *agos*PPI were selected as reference genes [21]. Primers were designed using NCBI Primer BLAST (https://www.ncbi.nlm.nih.gov/tools/primer-blast/index.cgi?LINK_LOC=BlastHome accessed on 22 July 2022 (Table S1), before being synthesized by Tsingke Biotechnology Co. (Beijing, China). The sequencing RNA (1 µg) was used to synthesize the first-strand cDNA (template) using a reverse transcription kit (Vazyme Biotech Co., Ltd., Nanjing, China) and then mixed into a 10 µL reaction system by using a ChamQ SYBR fluorescence quantitative kit (Vazyme Biotech Co., Ltd., Nanjing, China). Fluorescence quantitative conditions were 95 °C for 3 min, followed by 40 cycles of 10 s at 95 °C and 30 s at 60 °C. A melting curve analysis was conducted to verify the specificity of amplification. The relative expression levels were calculated using the 2^−ΔΔCT^ method [22].

### 2.7. Statistical Analysis

All data were analyzed using SPSS 25.0 (SPSS Inc., Chicago, IL, USA). Data were analyzed by one-way analysis of variance (ANOVA) using Tukey HSD post hoc multiple paired statistical comparisons (*p* < 0.05) to determine fecundity under four photoperiods. For some of the data with uneven variance, Kruskal–Wallis test analysis was used. Independent sample *T*-tests (*p* < 0.05) were used to compare fecundity between continuous treatment in 8:16 light–dark cycles and switching to a 12:12 light–dark cycle treatment. Data were presented as the mean ± standard error (SE). Charts were generated using GraphPad Prism 6.0 (GraphPad Software Co., Ltd., La Jolla, CA, USA)

## 3. Results

### 3.1. Fecundity of Cotton Aphid

**Figure 1:**
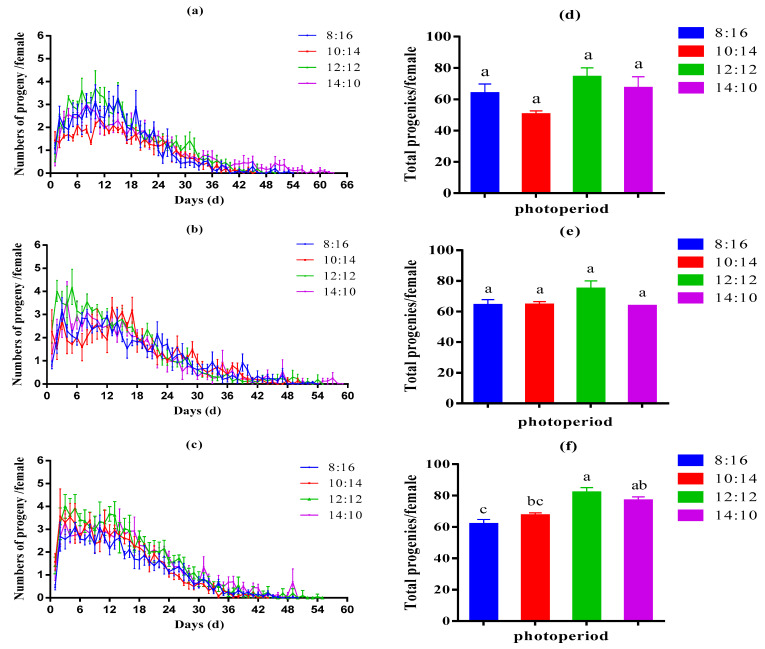
The numbers progeny per female per day (**a**–**c**) and total progenies per female (**d**–**f**) of first, third, and fifth generation cotton aphids under 8 L:16 D, 10 L:14 D, 12 L:12 D, and 14 L:10 D light–dark cycles. The results were represented as mean ± SE. Various letters above the bars indicate statistically significant differences between photoperiods (Tukey’s post hoc test; *p* < 0.05).

Photoperiod significantly affected the progeny numbers of aphids. The first, third, and fifth generations of aphids all grew under an L:D 12:12 photoperiod and had the highest reproduction curve, with a maximum number of daily maximum progenies of 3.71 (±0.77) per female for the first generation, 4.20 (±0.76) per female for the third and 4.00 (±0.52) per female fifth generations. While the 10:14 photoperiod treatment for the first generation (2.36 ± 0.38 per female), the 8:16 photoperiod treatment for the third generation (3.21 ± 0.51 per female), and the fifth generation (3.11 ± 0.20 per female) produced the fewest daily maximum progenies under various photoperiods (Figure 1a–c). Total progenies per aphid were highest at 12:12 photoperiod (the first generation: 73.96±6.08, the third generation: 74.54 ± 5.43, the fifth generation: 81.54 ± 3.45 per female). In addition, the lowest total progenies per aphid were recorded in the 10:14 photoperiod treatment for the first generation (50.05 ± 2.61 per female), the 8:16 photoperiod treatment for the third generation (63.86 ± 3.82 per female), and the fifth generation (61.55 ± 3.19 per female). In particular, the effect of photoperiod on total aphid progeny production reached significant levels in the fifth generation. In addition, aphid fecundity increased with increasing light duration and decreased beyond 12 h day length (Figure 1d–f).

### 3.2. Photoperiod Conversion Test

**Figure 2:**
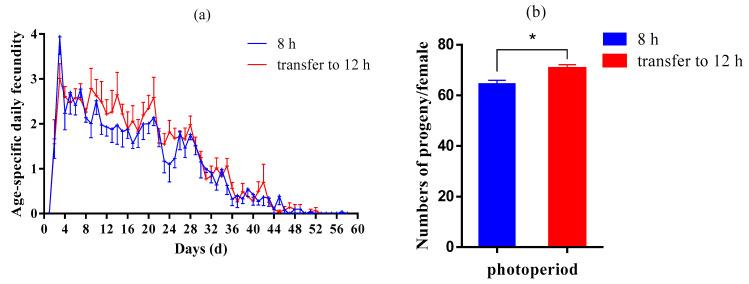
Age-stage-specific fecundity (**a**) and total fecundity per female (**b**) of aphids maintained under 8:16 light–dark cycles until the seventh generation and transferred to 12:12 light–dark cycles from the fifth generation. The results were represented as mean ± SE. * Indicates a significant difference between two photoperiods (*T*-test; *p* < 0.05).

We selected the fifth generation of aphids under a photoperiod of 8:16 and divided them into two treatments: continuous short light (8:16) and shifting to long light (12:12). After two generations, the reproduction curves of aphids under two photoperiodic conditions are shown in Figure 2a,b). Aphids that moved to 12 h light conditions had higher daily aphid production than those that remained in 8 h light conditions. The cumulative number of progenies produced by the aphids shifted to 12:12 (70.53 ± 1.57 per female), significantly higher than that, which was always at 8:16 (64.37 ± 1.60 per female).

### 3.3. Transcriptome Analysis

**Figure 3:**
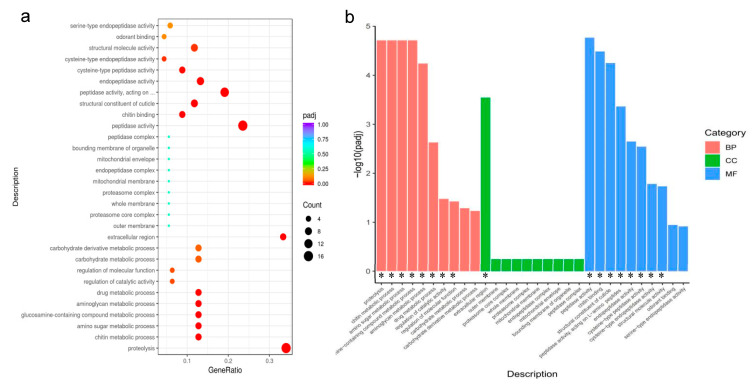
(**a**,**b**): GO enrichment of the DEGs. BP, biological process; CC, cellular component; MF, molecular function, using padj < 0.05 as the threshold for significant enrichment. * Indicates that DEGS is significantly enriched in this pathway.

To explore the transcriptomic response in the *A. gossypii* to two light–dark cycles, six cDNA libraries (three biological replicates) were constructed. In all tested samples, the base error rate was 0.03%; the Q20 value was in the range of 97.41~97.71%; the Q30 value was in the range of 92.59~93.28%, and the GC content was in the range of 34.21~40.35% (Table 1). This indicates that the construction quality of the sequencing library is good, and the data obtained by sequencing are accurate and reliable. A total of 170 DEGs under 8 h light compared to 12 h light (padj < 0.05). Among these DEGs, 123 downregulated and 47 upregulated were detected in comparison to 12 h light treatment (Figure 3a).

### 3.4. GO Analysis of DEGs

The functions of DEGs can be divided into three aspects: BP (biological processes), CC (cellular components), and MF (molecular functions). In 8 h light compared to 12 h light, 8 BP, 1 CC, and 8 MF catalogs were significantly aggregated (padj < 0.05 were considered significantly enriched). In BP, DEGs were enriched for proteolysis, chitin metabolic process, amino sugar metabolic process, glucosamine-containing compound metabolic process, aminoglycan metabolic process, drug metabolic process, regulation of catalytic activity, and regulation of molecular function. In CC, the DEGs were enriched in the extracellular region. In MF, GO terms mainly were involved in peptidase activity, chitin binding, structural constituent of the cuticle, peptidase activity, endopeptidase activity, cysteine-type peptidase activity, cysteine-type endopeptidase activity, and structural molecule activity. In Figure 2, we list the top 10 categories of the three GO terms. Most DEGs were downregulated in the short light treatment (Figure 3a,b).

### 3.5. KEGG Enrichment Analysis of DEGs

**Figure 4:**
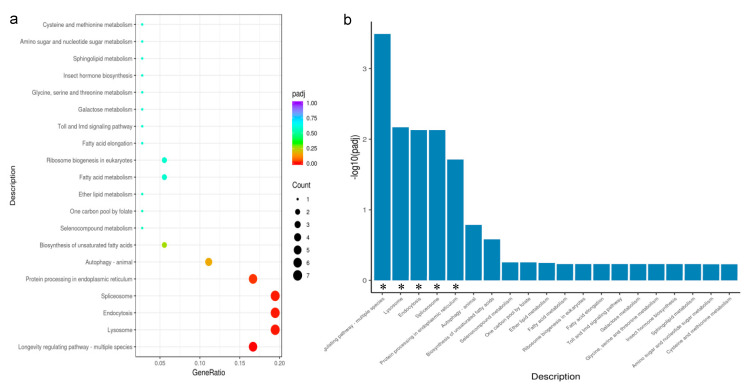
(**a**,**b**): Scatter and bar Chart of KEGG enrichment analysis of DEGs, using padj < 0.05 as the threshold for significant enrichment. * Indicates that DEGS is significantly enriched in this pathway.

To explore the pathways that may be involved in the KEGG pathway under two photoperiods, we performed KEGG enrichment analysis of DEGs under 8 h light and 12 h light. The significant KEGG enrichment pathways were classified into five categories: longevity regulating pathway-multiple species, lysosome, endocytosis, spliceosome, and protein processing in the endoplasmic reticulum. All genes were downregulated after 8 h light compared to 12 h light, but two genes associated with endocytosis and spliceosomes were upregulated, respectively (Figure 4a,b).

### 3.6. RT-qPCR Validation

**Figure 5:**
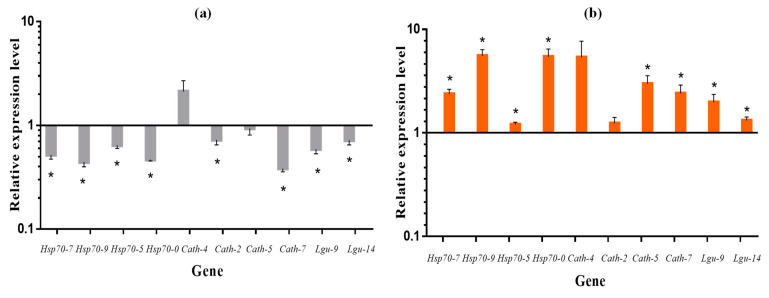
The relative expression levels of candidate genes in (**a**) the fifth generation *Aphis gossypii* between 8:16 and 12:12 (12:12 treatment as control, expression = 1) light–dark cycles; as well as (**b**): *Aphis gossypii* at 8:16 light–dark cycles until the seventh generation (8:16 treatment as control, expression = 1) and transferred to 12:12 light–dark cycles from the fifth generation. * Indicates a significant difference in the relative expression levels of genes (*T*-test; *p* < 0.05).

Next, we performed RT-qPCR on cotton aphid Hsp70 family genes, cathepsin B-like and legumain-like genes with large fold differences. A total of ten genes, including four Hsp genes, four cathepsin B-like and two legumain-like genes, were verified (Table 2). The results showed that the expression of all genes, except *Cath-4*, was lower at 8 h day length than at 12 h day length. The expression of *Hsp70-7*, *Hsp70-9*, *Hsp70-5*, *Hsp70-0* (Hsp70 family genes), and *Lgu-9*, *Lgu-14* (legumain-like genes) was significantly downregulated under short daylight. Of the four cathepsin B-like genes, the expression of *Cath-2* and *Cath-7* was significantly downregulated at short day lengths, while the expression of *Cath-4* and *Cath-5* was not significantly different compared to a 12 h of day length (Figure 5a). RT-qPCR results verifying the expression trends of cotton aphid differential genes between two photoperiods (L:D 8:16, 12:12) were generally consistent with the transcriptome results (Table 2). This indicated that the transcriptomic data were reliable. After transferring cotton aphids five generations under short daylight conditions to two generations under long light, the expression of all ten genes showed upregulation, compared to cotton aphids maintained under short light for seven generations. The expression of all genes, except for *Cath-4* and *Cath-2*, reached significant levels compared to the seven generations reared under short daylight (Table 2, Figure 5b).

## 4. Discussion

Photoperiod significantly influenced the reproduction of cotton aphids. Reproduction showed an increasing and then decreasing trend with the increase in daylight, reaching a maximum at the 12:12 photoperiod, indicating that the 12:12 light–dark cycles are the most favorable photoperiod for cotton aphids to produce progenies. You reported that the survival rate, adult male and female longevity, reproduction, and hatching rate of *Eocanthecona furcellata* larvae increased and then decreased with increasing light time, and the optimum growth and development photoperiod was considered to be 16L:8D [23]. Insects such as *Athetis lepigone* [24] *Agasicles hygrophila* [25] and *Apolygus lucorum* [26] were similar to our findings.

In the KEGG enrichment analysis, it was found that cotton aphids under short sunlight were significantly enriched by the “Longevity regulating pathway-multiple species”, “Lysosome”, “Endocytosis”, “Spliceosome” and “Protein processing in endoplasmic reticulum” and so on. HSP70 family genes dominated the pathways “Longevity regulating pathway-multiple species,” “Endocytosis”, “Spliceosome”, and “Protein processing in the endoplasmic reticulum”, with “Endocytosis” related to metabolism and “Spliceosome” related to transcription and translation. This indicates that HSP70 family genes play an important role in photoperiodic changes. Previous studies have shown that the expression of the heat stress protein HSP gene is significantly elevated in a variety of insects under heat stress conditions [27,28,29]. However, in recent years, photoperiodic changes have also been identified that can cause the expression of HSP70 family genes. Baykalir et al. showed that photoperiodic alterations would affect HSP70 levels in the heart and lungs of broilers [30]. Trionnaire et al. performed a transcriptional analysis of pea aphids (*Myzus persicae)* under natural conditions in summer (long daylight) and early fall (short daylight) and revealed that heat shock-associated proteins were downregulated in the fall, with a 7–8-fold downregulation in two transcripts [31]. Heat shock proteins of pea aphids may be downregulated in response to temperature changes, but this does not rule out the significant but often overlooked effect of a concurrent shortening of the photoperiod. Our study revealed for the first time that a single photoperiodic change also induced responses in the HSP70 family of genes in cotton aphids. Several studies have confirmed the association of the HSP70 gene with reproduction in insects, such as HSP70-4 (GG4264) of *Drosophila* [32] is essential for healthy egg formation and HSP70-5 [33] is associated with female fecundity. You (2017) used the feeding method for RNA interference with the heat shock protein HSP70 of *N. lugens* under Jinggangmycin treatment (which stimulates egg laying and improves heat tolerance), resulting in reduced oviposition, longer pre-oviposition period and shorter oviposition period at high temperatures (34 °C) [34]. Chen (2021) studied the genes and functions of the HSP family of *Nilaparvata lugens*, revealing that HSP70 has important functions in ovarian development, embryonic development, and egg-stage heat tolerance [35]. The “Lysosome” pathway is dominated by cathepsin B-like and legumain-like family genes. Cathepsin is involved in many important life activities in living organisms. Studies in mammals have shown that Cathepsin is mainly found in the lysosomes of normal cells and particularly expressed in malignant cells [36,37], and can also be involved in apoptosis [38]. Additionally, it is prevalent in oviparous creatures such as fish and insects, where it hydrolyzes yolk proteins to provide crucial amino acids for embryonic development. However, the function of legumain-like genes in insect reproduction is not yet known.

GO enrichment analysis revealed that cotton aphids under short sunlight had a large number of differential genes enriched in “proteolysis”, “metabolic process”, “peptidase activity” and “structural molecule activity” pathways. Among the “metabolic process” related pathways are chitin metabolic, amino sugar metabolic, glucosamine-containing compound metabolic, aminoglycan metabolic and drug metabolic process was dominated by Chitin binding Peritrophin-A domain family genes. Min et al. reported that the function of chitin-binding peritrophin is mainly to facilitate the formation of chitin–protein structures (Asiatic honeybee *Apis cerana)* [39]. We speculate that some structural changes in the cotton aphid epidermis are likely to occur during the change in photoperiod.

RT-qPCR revealed that the expression of the Hsp70 family genes, legumain-like genes, and *cath837 of* cathepsin B-like and were more sensitive to the photoperiodic transition in both validations. These genes may play an important role in photoperiod change and regulate the reproduction of *A. gossypii*, but their specific functions remain unclear. We will further verify the specific functions of these genes by RNAi or reliable methods.

This experiment was carried out under constant temperature and humidity conditions and the effects of photoperiod on cotton aphid reproduction were discussed and clarified, without considering the combined effect of temperature and humidity and photoperiod. In addition, the biotype of the aphid studied in this experiment is the cotton type, which has been reported in previous studies to overwinter as eggs through the production of sexual aphids [40], whereas in this study no sexual aphids were induced in several trials. This leads to the assumption that *A. gossypii* have anholocyclic populations. Liu’s study demonstrates that latitude and longitude are also important factors influencing the reproductive mode of *A. gossypii* [41]. Other factors influencing the shift in aphid reproduction will be investigated in future studies.

## 5. Conclusions

The present results show that photoperiod affects the reproduction of *A. gossypii*. In general, daylight extension is more conducive to the laying young of *A. gossypii*, especially during the L: D 12:12 photoperiod. A total of 170 DEGs were identified between the 8 h short light and 12 h long light treatments via comparative transcriptomic analysis. In addition, 123 downregulated and 47 upregulated genes were identified in aphids under two photoperiods. The signaling pathways potentially involved under different photoperiod treatments were obtained including longevity regulating pathway-multiple species, spliceosome, metabolic process, peptidase activity and degradation, and so on. Most of the genes involved in these pathways were heat shock protein HSP70 gene family, legumain-like, and cathepsin B-like. The expression levels of candidate genes were validated by RT-qPCR soon afterward, demonstrating that genes involved in these pathways play an important role in photoperiod change. This study filled in the gaps in the research on the molecular mechanism for that photoperiod change regulates the reproduction of *A. gossypii,* and further studies of the functions of candidate genes by RNAi methods will help to develop genetic control strategies against this pest by disrupting its reproduction.

## Figures and Tables

**Table 1:** Quality control result of sequencing data of Aphis gossypii under 8:16 and 12:12 light–dark cycles.

Sample	Raw_Reads	Clean_Reads	Clean_Bases	Error_Rate	Q20 (%)	Q30 (%)	GC_pct
a8h_1	40475538	38483248	5.77 G	0.03	97.55	92.81	34.21
a8h_2	44987626	43379616	6.51 G	0.03	97.46	92.68	36.99
a8h_3	45342310	43130526	6.47 G	0.03	97.65	93.12	39.19
b12h_1	44153910	41905684	6.29 G	0.03	97.41	92.59	38.03
b12h_2	47541074	44842714	6.73 G	0.03	97.71	93.28	40.35
b12h_3	44519246	42452724	6.37 G	0.03	97.52	92.82	37.47

GC_pct: Percentage of G and C of the four bases in clean reads.

**Table 2:** The relative expression levels of candidate genes of the fifth generation *Aphis gossypii* under 8:16 vs. 12:12 light–dark cycles; and *Aphis gossypii* under continuous 8:16 vs. transfer from 8:16 to 12:12 light–dark.

Gene_id	8 h vs. 12 h (log2FoldChange)				Remain at 8 h vs. Transfer to 12h (log2FoldChange)	Tf_Family
	Pvalue	Padj	RNA-seq	RT-qPCR	RT-qPCR	
114133077(HSP-70-7)	0.05	1.00	−2.20	−0.97 ± 0.12	1.20 ± 0.16	HSP70
114122099(HSP-70-9)	0.04	1.00	−2.26	−1.21 ± 0.15	2.39 ± 0.22	HSP70
114119569(HSP-70-5)	0.00	1.00	−2.18	−0.66 ± 0.09	0.26 ± 0.07	HSP70
114132056(HSP-70-0)	0.01	1.00	−1.88	−1.10 ± 0.05	2.30 ± 0.29	HSP70
114129224(cB-4)	0.02	1.00	−0.93	0.60 ± 0.61	1.72 ± 0.62	Peptidase_C1
114118922(cB-2)	0.01	1.00	−1.20	−0.51 ± 0.17	0.24 ± 0.19	Peptidase_C1
114121295(cB-5)	0.04	1.00	−1.08	−0.16 ± 0.19	1.43 ± 0.29	Peptidase_C1
114121837(cB-7)	0.01	1.00	−0.55	−1.40 ± 0.10	1.13 ± 0.27	Peptidase_C1
114125009(le-9)	0.02	1.00	−0.44	−0.79 ± 0.14	0.85 ± 0.28	-
114125014(le-14)	0.02	1.00	−3.24	−0.51 ± 0.13	0.38 ± 0.11	-

## Data Availability

The data presented in this study are available on request from the first author.

## Supplementary Materials

The following supporting information can be downloaded at: https://www.mdpi.com/article/10.3390/insects13121105/s1, Table S1: Primers used in RT-qPCR.
