# Supplementary material for: Reproduction Evaluation and Transcription Analysis of Aphis gossypii under Various Photoperiods
Source: Insects. 2022 Nov 30;13(12):1105. doi: 10.3390/insects13121105 (PMC9787806; doi:10.3390/insects13121105)
Supplement: Supplementary file 1 [file insects-13-01105-s001.zip › insects-1990995-SI.pdf]

Supplement Table S1：Primers used in RT-qPCR.

Gene	5' –3'
DIMT F	AGCCACTAACCTAACATC
DIMT R	TCAAGGAATGGTAGAGAAG
PPI F	GTCTTCATTACAGTCTATGG
PPI R	ATTGAGTGGTAGATGAGTT
HSP-70-7 F	TCGACGAAGCAATGGTTGGA
HSP-70-7 R	TTCCGTGTTGCCAGATACCC
HSP-70-9 F	GGAACGCGGGTAAACAGGAA
HSP-70-9 R	CGAGAACCGGGGAAACCAC
HSP-70-5 F	AGTTGTGCGCGGATCTGTT
HSP-70-5 R	GGCTACAGCTTCATCGGGATTT
HSP-70-0 F	CAGTGCGACCGAAACACATTA
HSP-70-0 R	CGCAATAACGCACAATTCGATG
cB-4 F	CCGAGCACACCAAAGGAAGA
cB-4 R	TTCCGAAAGCCCAGCAAGAA
cB-2 F	GGCCCTATCAACCGCTTCTG
cB-2 R	CCGTTACATCCGTCACCACA
cB-5 F	TTGTGGAAGTTGTTGGGCCT
cB-5 R	CCTCCAGTGACAAGACCGTG
cB-7 F	CACGGGCCTACAACTGCTAT
cB-7 R	TGAACGCCTCTGTACACACC
le-9 F	CGATGGTTGCGAAGGCTAGA
le-9 R	AACCGATCCAAAGCGTAGGG
le-4 F	TCGTGGAAGGTGCAGAAATCA
le-4 R	ACCGACAACTTACCGTGAGC
